# Supplementary material for: Identification of Elite R-Gene Combinations against Blast Disease in Geng Rice Varieties
Source: Int J Mol Sci. 2023 Feb 16;24(4):3984. doi: 10.3390/ijms24043984 (PMC9960461; doi:10.3390/ijms24043984)
Supplement: Supplementary file 1 [file ijms-24-03984-s001.zip › Figure S1-S7.pdf]

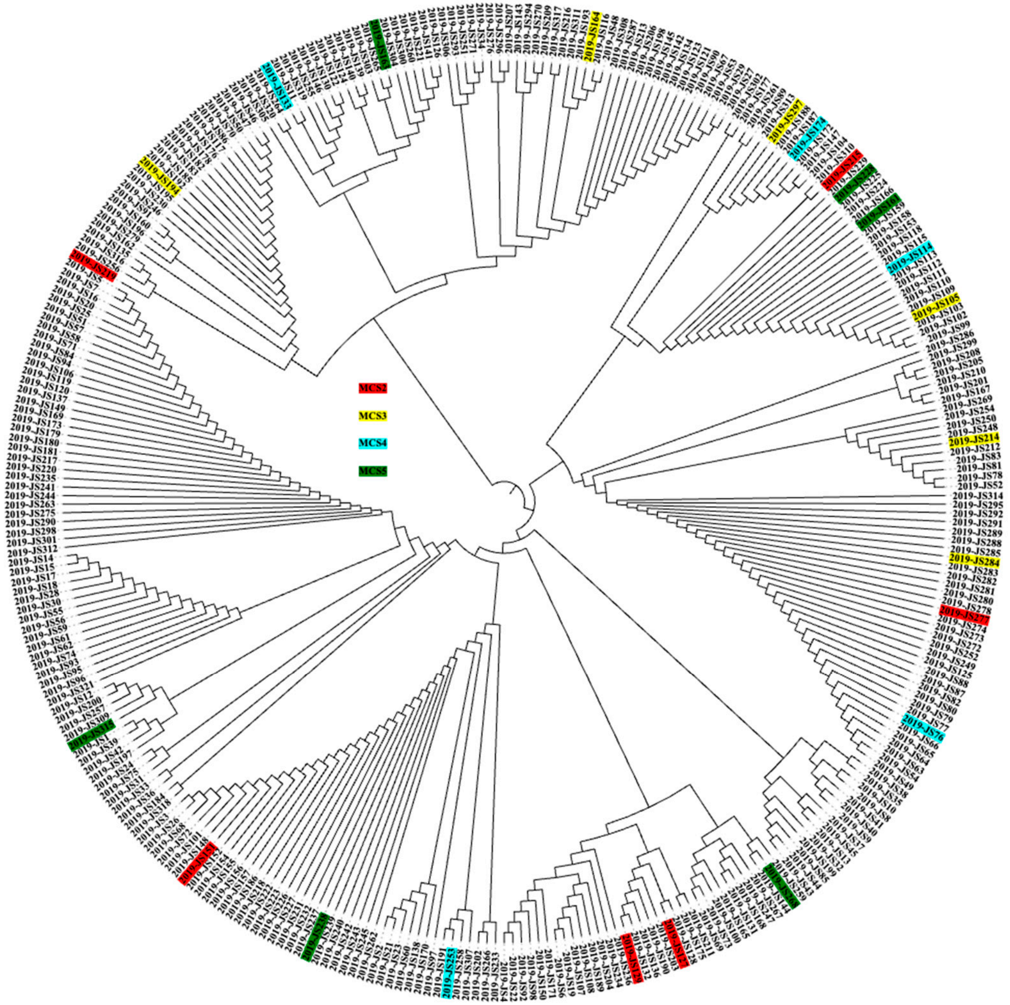

Figure S1. Clustering analysis of 321 isolates using the genotyping data of 10 characterized *avirulence* genes.

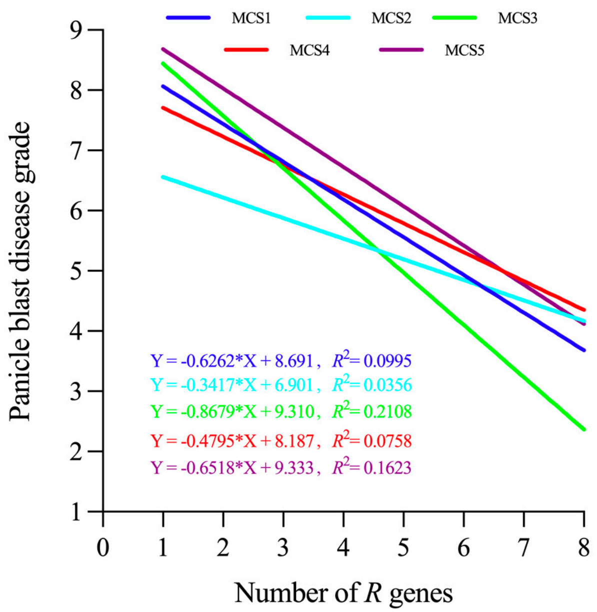

Figure S2. Regression analysis of the panicle blast disease grades in each MCS inoculation and the number of *R* genes in each variety.

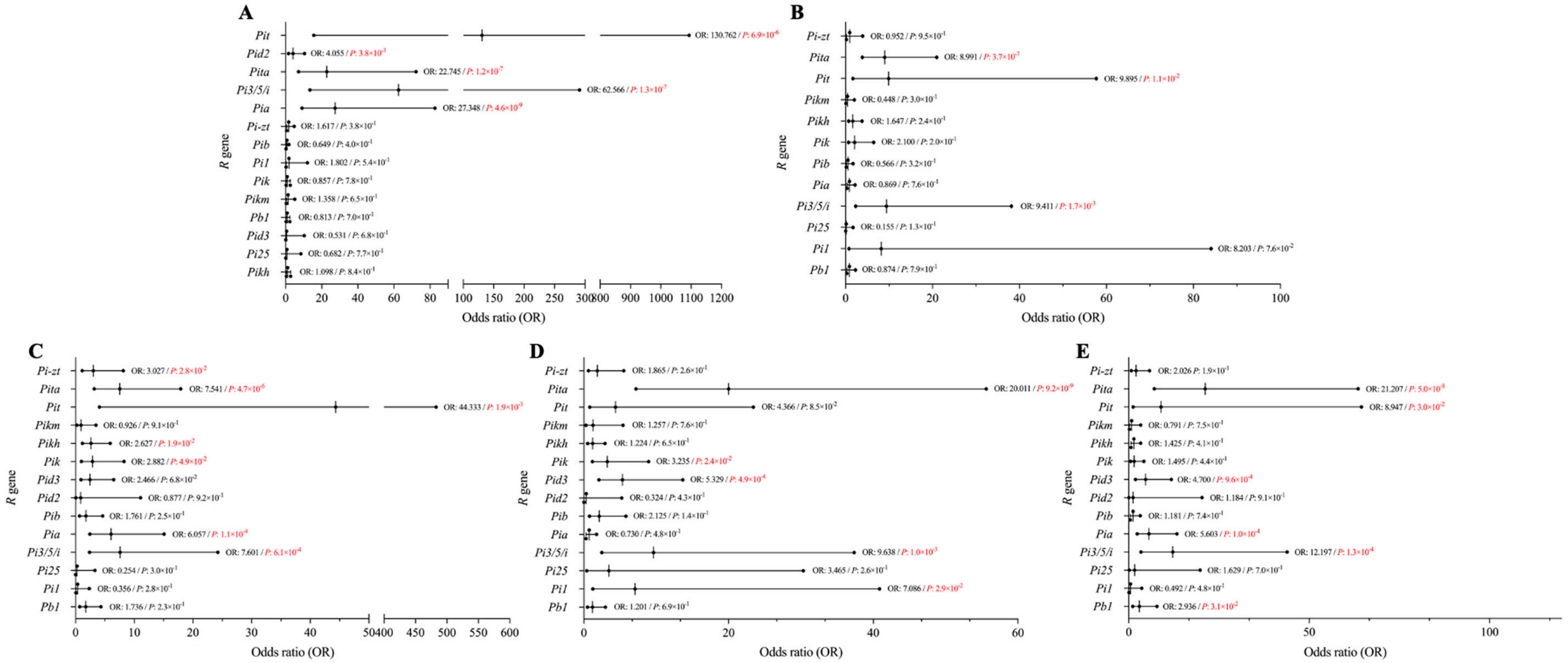

Figure S3. (**A**)-(**E**) Multinomial logistics regression analysis between the genotypes of *R* genes and disease grades of the tested varieties in different MCS inoculations. Red texts indicate that the contribution of the corresponding *R* genes to panicle blast resistance has reached a significant level ($P < 0.05$).

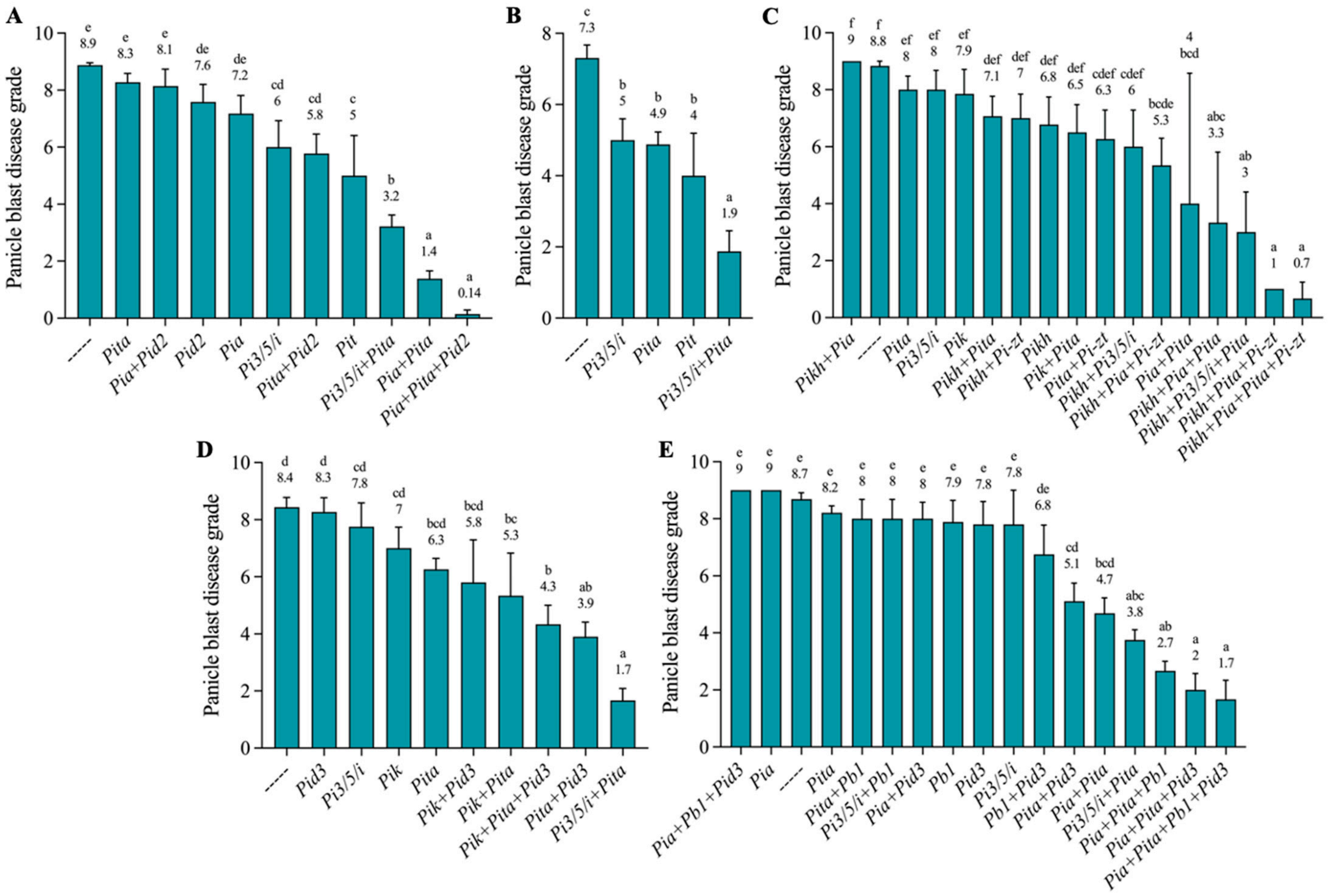

Figure S4. (**A**)-(**E**) Comparison of the disease grades of the varieties containing different elite *R* genes or *R*-gene combinations for panicle blast resistance in different MCS inoculations. Different lowercase letters indicate significant differences according to *Duncan*'s multiple range tests ($P < 0.05$).

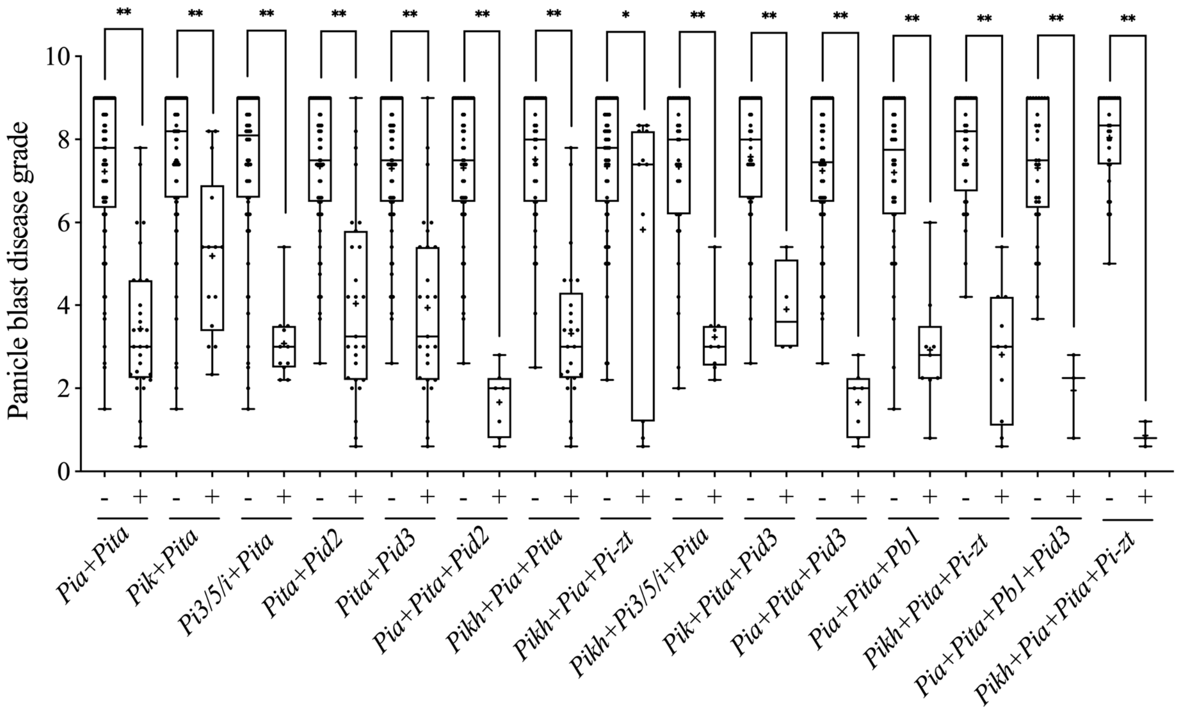

Figure S5. Comparisons of the panicle blast disease grades in 5 MCS inoculations between elite *R*-gene combinations and corresponding control. “-” and “+” denote the varieties with or without the corresponding gene combinations. **: $P < 0.01$ and *: $P < 0.05$ by Student’s two-sided *t*-test.

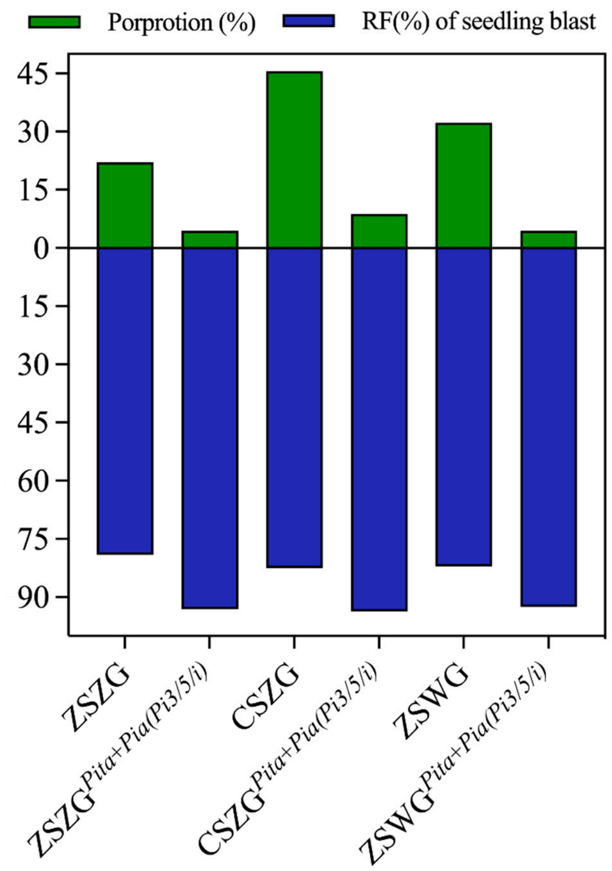

Figure S6. Application of *Pita+Pia* (*Pi3/5/i*) in tested varieties for seedling blast resistance evaluation with different ecotypes. Green columns denote the proportion of different ecotypes among the tested varieties and blue columns represent the average RF of seedling blast of different ecotypes.

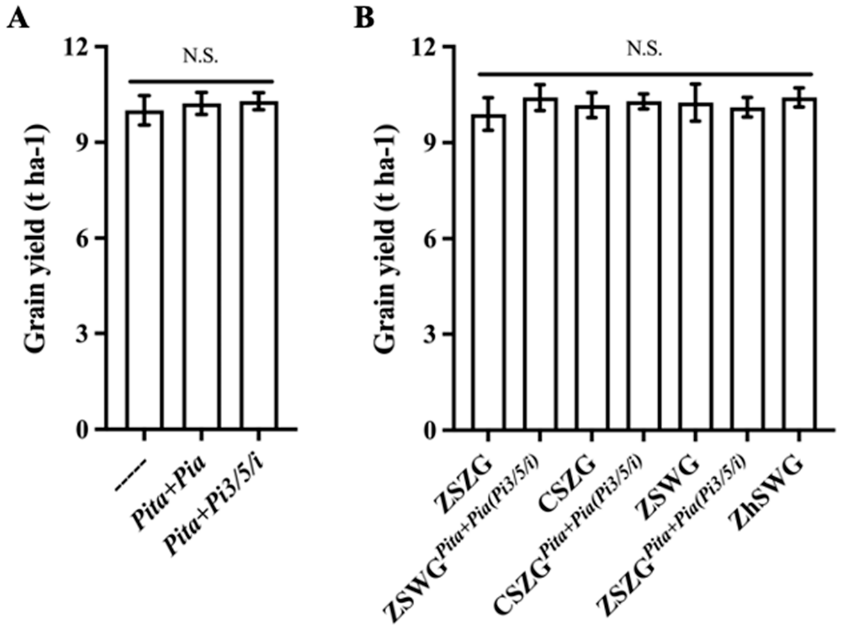

Figure S7. Analysis of the grain yields for *Pita*+*Pia* (*Pi3/5/i*) in (**A**) 190 tested varieties and (**B**) the varieties with different ecotypes. N.S. indicates no significant difference.
